# In Vitro Determination of Drug Transfer from Drug-Coated Balloons

**DOI:** 10.1371/journal.pone.0083992

**Published:** 2013-12-31

**Authors:** Anne Seidlitz, Nadine Kotzan, Stefan Nagel, Thomas Reske, Niels Grabow, Claus Harder, Svea Petersen, Katrin Sternberg, Werner Weitschies

**Affiliations:** 1 Institute of Pharmacy, Center of Drug Absorption and Transport, University of Greifswald, Greifswald, Germany; 2 Institute for Biomedical Engineering, University of Rostock, Rostock, Germany; 3 Biotronik SE & Co. KG, Erlangen, Germany; University of California Merced, United States of America

## Abstract

Drug-coated balloons are medical devices designed to locally deliver drug to diseased segments of the vessel wall. For these dosage forms, drug transfer to the vessel wall needs to be examined in detail, since drug released into the blood is cleared from the site. In order to examine drug transfer, a new in vitro setup was developed combining the estimation of drug loss during advancement to the site of application in a model coronary artery pathway with a hydrogel compartment representing, as a very simplified model, the vessel wall. The transfer of fluorescent model substances as well as the drug paclitaxel from coated balloons to the simulated vessel wall was evaluated using this method. The model was suitable to quantify the fractions transferred to the hydrogel and also to qualitatively assess distribution patterns in the hydrogel film. In the case of fluorescein sodium, rhodamin b and paclitaxel, vast amounts of the coated substance were lost during the simulated passage and only very small fractions of about 1% of the total load were transferred to the gel. This must be attributed to good water solubility of the fluorescent substances and the mechanical instability of the paclitaxel coating. Transfer of the hydrophobic model substance triamterene was however nearly unaffected by the preliminary tracking procedure with transferred fractions ranging from 8% to 14%. Analysis of model substance distribution yielded inhomogeneous distributions indicating that the coating was not evenly distributed on the balloon surface and that a great fraction of the coating liquid did not penetrate the folds of the balloon. This finding is contradictory to the generally accepted assumption of a drug depot inside the folds and emphasizes the necessity to thoroughly characterize in vitro performance of drug-coated balloons to support the very promising clinical data.

## Introduction

Drug-coated balloons (DCB, in this context referring to coated angioplasty catheter balloons) are drug-device combination products designed to deliver drug locally to certain diseased segments of the vessel wall. Compared to drug-eluting stents (DES) which possess a similar field of indications, potential advantages of DCB are homogenous drug transfer to the treated vessel portion, rapid drug release providing the desired antiproliferative action with little impact on long-term healing and possibly shorter continuation of dual antiplatelet therapy, and absence of remaining polymer implants which may cause chronic inflammation and late thrombosis [1]. The current drug of choice is paclitaxel (PTX) whose physicochemical properties seem to make the drug substance most suitable for this application [2], [3]. Different PTX formulations have been used including drug only coatings, as well as coatings including small fractions (typically 10%) of different additives, such as iopromide, urea, shellac, butyryl trihexyl citrate or a combination of polysorbate and sorbitol [3]–[5]. PTX is typically applied onto the balloon surface at a concentration of 3 µg/mm^2^ [4].

Even though a number of different devices are available in different countries, with at least ten CE-marked DCB in Europe, the clinical applicability of the technology is not yet fully understood [6]. While a fairly large number of reported clinical studies including different types of lesions, patients, and locations has not been able to resolve the existing uncertainties [6], hardly any literature on in vitro characterization of these devices has been published. In comparison to some traditional dosage forms, the examination of drug release into stirred media will not answer the questions associated with this type of delivery, since only drug transferred to the vessel wall will be able to impart the desired effect, while drug released into the blood will be cleared from the site. Many assumptions regarding drug transfer from DCB to the vessel wall exist, which have not been properly evidenced by published data until to-date. Therefore, it was the aim of this study to establish a method for in vitro examination of drug transfer from DCB to a simulated vessel wall under consideration of some of the conditions prevailing during in vivo application, and to test the release from balloons coated with model substances of different physicochemical properties, as well as the clinically used drug PTX.

## Materials and Methods

### Materials

Fast exchange PTCA balloon catheters (design Pantera Lux, 3.5×20 mm, without the PTX-containing coating as commercially available) were provided by Biotronik AG, Switzerland. Paclitaxel (PTX) was obtained from Cfm Oscar Tropitzsch e.K., Germany. Fluorescent model substances rhodamine B (RHO), triamterene (TRI) and fluorescein sodium (FLU) were purchased from Sigma Aldrich Chemie GmbH, Germany. Sodium alginate was obtained from Fagron GmbH & Co. KG, Germany. Solvents and all other substances used were of analytical grade.

### Methods

#### Balloon coating

Balloons were coated with the fluorescent model substances FLU, RHO, TRI or the drug PTX. The water solubility and log P values of the model substances and drug are given in table 1 [7]–[15].

**Table 1:** Physicochemical properties of the used model substances/drug.

	Solubility in water (mg/mL)	log P
Fluorescein sodium	100–1000 [7]	−1.52 [8]
Rhodamin B	> 1000 [7]	2.30 [9]
Triamterene	0.0028 [10]	1.25 [11]
Paclitaxel	0.001 [12] 0.0004 [13]	4.4 [14] 3.96 [15]

A micro-pipetting technique, which is most commonly used for balloon coating [2], was applied for the coating process. For this purpose the folded balloon was manually rotated along its shaft and the solution or suspension of the respective substance was carefully applied onto the surface. It was observed in preliminary experiments that the visual coating distribution homogeneity could be improved when smaller droplets were applied onto the balloon surface. For this reason a Hamilton syringe (Hamilton Bonaduz AG, Switzerland, volume 25 µl, gastight # 1702) was used to apply drops of the coating liquid onto the surface of the folded balloon, especially along the crests of the folds to allow for the penetration of the liquid into the folds. Drying intervals were included in the coating process during which the tip of the balloon was moved up and down. In preliminary experiments different organic solvents were tested regarding their applicability for the coating process and the stability of the balloon foil consisting of a polyether block amide in the presence of the solvents (data not shown). The finally used coating liquids contained the model substance/drug at a concentration of approximately 7 mg/mL. Solvents were methanol for PTX, ethanol for FLU as well as RHO, and dichloromethane for TRI. Due to the fact that it was not possible to achieve the necessary concentration of dissolved TRI in the tested solvents, a fine TRI suspension in dichloromethane was used. 100 µL of the solutions or suspension were applied onto each balloon, resulting in a drug load of approximately 3 µg/mm^2^ (corresponding to a total drug load of approximately 700 µg). Drug carriers or additives were not used in this study. Coatings were inspected microscopically and imaged as described below. In addition, a FLU-coated balloon was separated from the catheter shaft after dry expansion and the top of the balloon folds were marked with a pen. The balloon foil was carefully cut open and photographed.

#### Model coronary artery pathway

**Figure 1:**
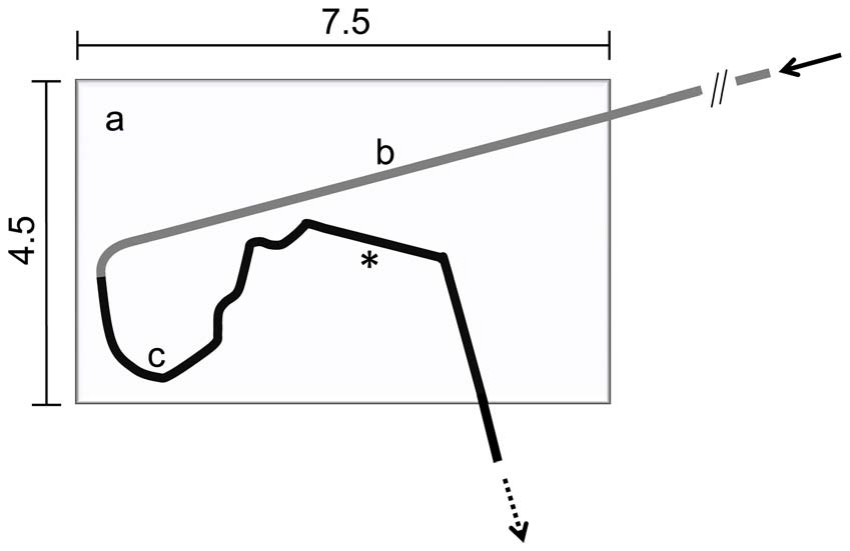
Schematic of the model coronary artery pathway. Model adapted from [17] with a) polymethacrylate frame, b) coronary guiding catheter, c) tube, asterisk marking balloon resting position, arrows marking the insertion point of the balloon through a hemostatic valve (continuous line) and the exiting point after passage of the model (broken line); measures in inches.

A previously described polymethacrylate model [16] adapted from ASTM standard F 2394-07 [17] was used to simulate the in vivo implantation procedure of DCB. A schematic of this model is given in figure 1. The model consists of three polymethacrylate plates into which a simulated course of a coronary artery is shaped. The first portion of the simulated pathway was equipped with a guiding catheter (Long Vista Brite Tip 5 F JL 3.5 LBT, Cordis Europe, Belgium). The proximal end of this catheter was connected to a hemostatic valve (Y-connector with rotating adapter and Tuohy-Borst valve, B. Braun Melsungen AG, Germany). This valve was connected to a media container via tubing (Tygon R3607 inner diameter 3.17 mm, VWR International, Germany) and a peristaltic pump (Reglo Digital, Ismatec, Germany). The distal end of the guiding catheter was connected leak-proof to another tubing (Tygon R3607 inner diameter 3.17 mm, VWR International, Germany) which lined the further length of the simulated arterial pathway. A coronary guide wire (Galeo M 0,014″, Biotronik AG, Switzerland) was placed in the simulated artery lumen through the hemostatic valve.

#### Model vessel wall compartment

Calcium alginate films were prepared as previously described [18]. In brief, sodium alginate solutions (3% m/m in purified water) were cast onto a glass plate by means of a doctor blade with a slot width of 500 µm. The solution was immediately gelled by topping with calcium chloride solution (6% m/m in purified water). After 10 min, excess liquid was removed and films were cut to approximately 5×8 cm rectangles. These rectangles were coiled around a stainless steel rod (diameter 3 mm) forming approximately 5 coils (layers) of film. The coiled shape was fastened and the stainless steel rod was removed thus forming a rolled up gel film cylinder (length 5 cm) with a central opening of 3 mm in diameter. The gel cylinders were used for drug transfer testing immediately after preparation.

#### Drug release and transfer testing

DCB were introduced into the guiding catheter of the model coronary artery pathway through the hemostatic valve which was then closed in order to prevent media loss. The DCB was advanced quickly along the guide wire until reaching the resting position (see asterisk figure 1, time required approximately 20 s). The peristaltic pump was started at beginning of the advancement of the DCB through the system, thus pumping phosphate buffered saline pH 7.4 (PBS), prepared according to Ph. Eur., at a flow-rate of 35 mL/min through the simulated coronary pathway. The DCB was subjected to the flowing media for a time span of 1 min. For TRI, a time span of 5 min was tested additionally. Media was collected at the outlet. At the ending of the intended perfusion time the media flow was terminated and the balloon was further advanced forward out of the model. The balloon was then inserted into the gel cylinder (see above) and expanded against the gel using an inflation device (Indeflator, Abbott Vascular, USA, filled with purified water) at a pressure of 8 atm for exactly 1 min. After the transfer time of 1 min the balloon was deflated and removed. Taking into account the diameter of the stainless steel rod used for film coiling and the diameter of the balloon, the balloon expansion procedure resulted in an “overstretch” of approximately 17%.

#### Imaging

Images of gel films were immediately obtained after FLU- and RHO-coated balloon expansion, removal and unwinding of the film using a digital camera (D 5100, Nikon GmbH, Germany). Images of a separated coated balloon foil were also obtained using the same equipment. Model substance coatings were examined by light microscopy (Axiovert 200, imaging via AxioCam HrC, both Carl Zeiss MicroImaging GmbH, Germany). In the case of TRI, gel films were imaged using a fluorescence microscope (BZ-8000 with BZ-Analyser, Keyence Deutschland GmbH, Germany λ_ex_ 360 nm, λ_em_ 460 nm). For the PTX-coated balloons, coating morphology was examined with a Philips XL 30 environmental scanning electron microscope (ESEM, Philips Electron Optics, Netherlands) operating in the ESEM mode with a water vapor pressure of 1.2 mbar. The accelerating voltage was set to 10 kV, the beam current to 11 µA and the working distance to 16.4 mm.

#### Sample preparation

For model substance quantification, gel films were transferred to glass containers and a suitable amount of 10-fold concentrated phosphate buffer solution pH 7.4 according to USP was added. These mixtures were stirred until the gel film had liquefied through the replacement of Ca^2+^-ions by monovalent ions thus reversing the gelling process. Care was taken to ensure that all model substance transferred to the gel film was dissolved prior to sampling. In the case of the hydrophobic TRI, the identical volume of methanol was added to ensure complete dissolution. Model substance content of diluted liquefied hydrogel films was determined fluorimetrically as described below.

PTX containing gel films were extracted in order to prepare PTX for high performance liquid chromatography (HPLC, see below). Three consecutive extractions were performed by adding a suitable amount of methanol to the gel samples and shaking (first extraction 5 min, second and third extraction 15 min). The liquid phase was removed, accurately weighed, diluted with PBS pH 7.4, centrifuged (centrifuge 5702 R, Eppendorf AG, Germany, 15 min, 3 G), and the PTX content was determined via HPLC. The gel was again extracted.

Perfusion media was collected at the end of the simulated arterial pathway. Since particle delamination from the DCB might have occurred during the perfusion, the perfusates of PTX and TRI-coated balloons were diluted with methanol prior to sampling in order to dissolve conceivably contained particulate matter. FLU and RHO are very soluble in water (see table 1) so that dilution was not necessary for these model substances. Clear solutions were obtained in all cases. For the 5 min perfusion experiments, the container for media collection was replaced with an empty container after each minute thus collecting 5 separate media fractions.

The amounts remaining on the balloons were determined after clipping of the catheter shafts and incubating the balloons in a suitable amount of solvent (PBS pH 7.4 for FLU, ethanol for RHO, and methanol for TRI and PTX).

In order to quantify the amounts remaining in the coronary pathway model, the system was rinsed thoroughly with a suitable amount of an appropriate solvent for the respective model substance/drug (PBS pH 7.4 for FLU, ethanol for RHO, and methanol for TRI and PTX). The mass of the rinse fluid was determined accurately.

In order to limit evaporation, all samples in organic solvents were diluted with the identical volume of PBS pH 7.4 prior to quantification.

#### Quantification

For quantification of the fluorescent model substances, 2×200 µL of each sample were transferred to a 96-well plate (cell culture test plate 96 F, TPP/Biochrom AG, Germany) and fluorescence intensity was measured against two standard calibration curves on the same microplate in the same sample matrix using a fluorescence reader (Varioskan Flash, Thermo Scientific, USA). Measurements were performed at the following wavelengths: FLU λ_Ex_ = 490 nm, λ_Em_ = 515 nm; RHO λ_Ex_ = 554 nm, λ_Em_ = 580 nm; TRI λ_Ex_ = 360 nm, λ_Em_ = 432 nm. Fluorescent substances and samples thereof were protected from light whenever possible throughout the experiments.

PTX content of the samples was determined using a HPLC system (Gerätebau Dr. Ing. Herbert Knauer GmbH, Germany) equipped with a degasser/gradient module (Manager 5000), mixing chamber, pump (Smartline 1000), autosampler (Smartline 3800), column oven (Jet Steam Oven) and a UV-detector (Smartline 2600). The used column (Chromolith FastGradient RP18e 50 – 2 mm) was kept at a temperature of 23°C throughout the separation. The chromatography was performed under isocratic conditions with a mobile phase consisting of acetonitrile/phosphate buffered saline solution 0.005 M pH 3.5 (50/50 v/v). The flow rate was set to 0.3 mL/min. UV detection was conducted at a wavelength of 230 nm. The injected sample volume was 20 µL. All measurements were performed in duplicate.

All data is presented as percentage of the sum of the detected amounts in all compartments for the respective DCB. Means of n = 3 ± standard deviation (SD) are reported. On average, the recovered substance amounted to more than 85% of the theoretical load (determined by the volume and concentration of the coating solution).

## Results

### Balloon coatings

**Figure 2:**
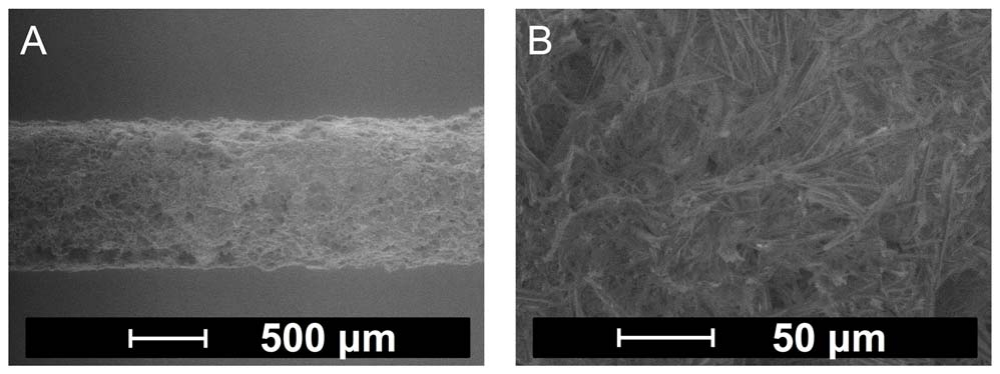
Morphology of PTX coating. Representative environmental scanning electron microscopic images of a folded paclitaxel-coated balloon in different resolutions.

Balloons were successfully coated with the substances and remained tightly folded during the coating process. Microscopic examination of the folded balloons yielded visual smooth coatings for FLU and RHO whereas TRI and PTX coatings were structured. Representative ESEM images of PTX-coated balloons are depicted in figure 2. Crystallized PTX needles can be observed on the surface (figure 2B).

**Figure 3:**
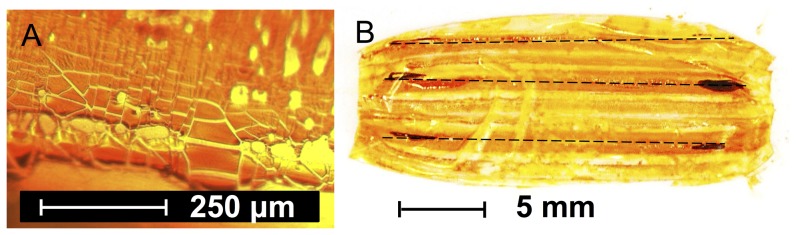
Distribution of FLU coating. Representative light microscopic image of the coating (A) and photograph of a separated balloon foil of a fluorescein sodium-coated balloon after dry expansion (B), broken lines indicating the location of the crests of the folds in the deflated state.

After balloon expansion, inhomogeneities in the initially smooth FLU and RHO coatings could be detected. Exemplary images of FLU coatings are given in figure 3. On the one hand, fine segmentation of the intensively colored regions was observed (figure 3 A). This fracturing of the coating most likely occurred during balloon expansion. Distinct particle delamination was not observed during expansion. On the other hand, differences between the different regions of the balloon were observed, as visible in the image of the separated balloon foil (figure 3 B). The coloration was most intense in the crest sections of the balloon folds.

### Drug release and transfer testing

**Figure 4:**
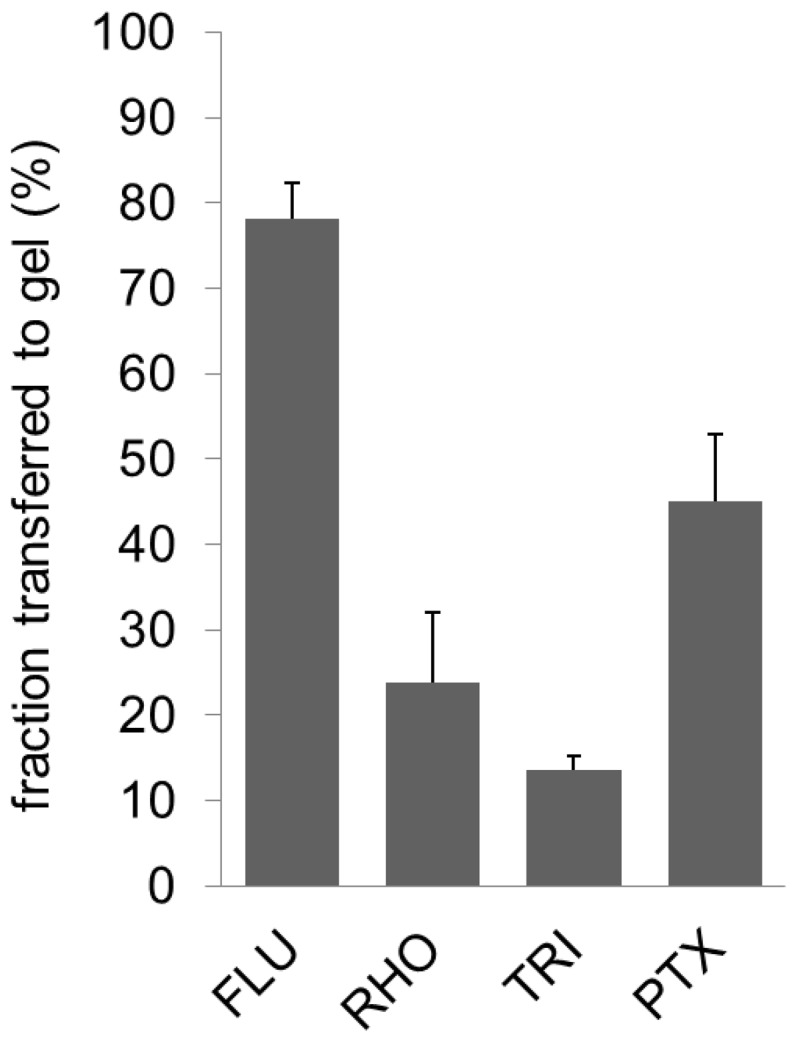
Drug transfer rate without simulated passage. Substance transfer from coated balloon to the gel during 1 =  fluorescein sodium, RHO  =  rhodamine B, TRI  =  triamterene, PTX  =  paclitaxel, means of n = 3±SD.

In a first set of experiments, drug transfer from DCB was evaluated without prior advancement through the model coronary pathway. Thus, the balloons were neither in contact with perfusion media nor subjected to mechanical stress prior to expansion into the gel film. The results of the model substance/drug content of the gel samples are depicted in figure 4. The greatest fractional drug transfer was detected for FLU with a transfer rate of 78%±4%. The fraction of PTX transferred to the vessel wall amounted to 45%±8% whereas in the case of RHO and TRI only 24%±8% and 14%±2% were detected in the gel compartment, respectively. The non-transferred fraction represents the fraction remaining on the balloons after expansion and deflation in these experiments (difference to 100%).

**Figure 5:**
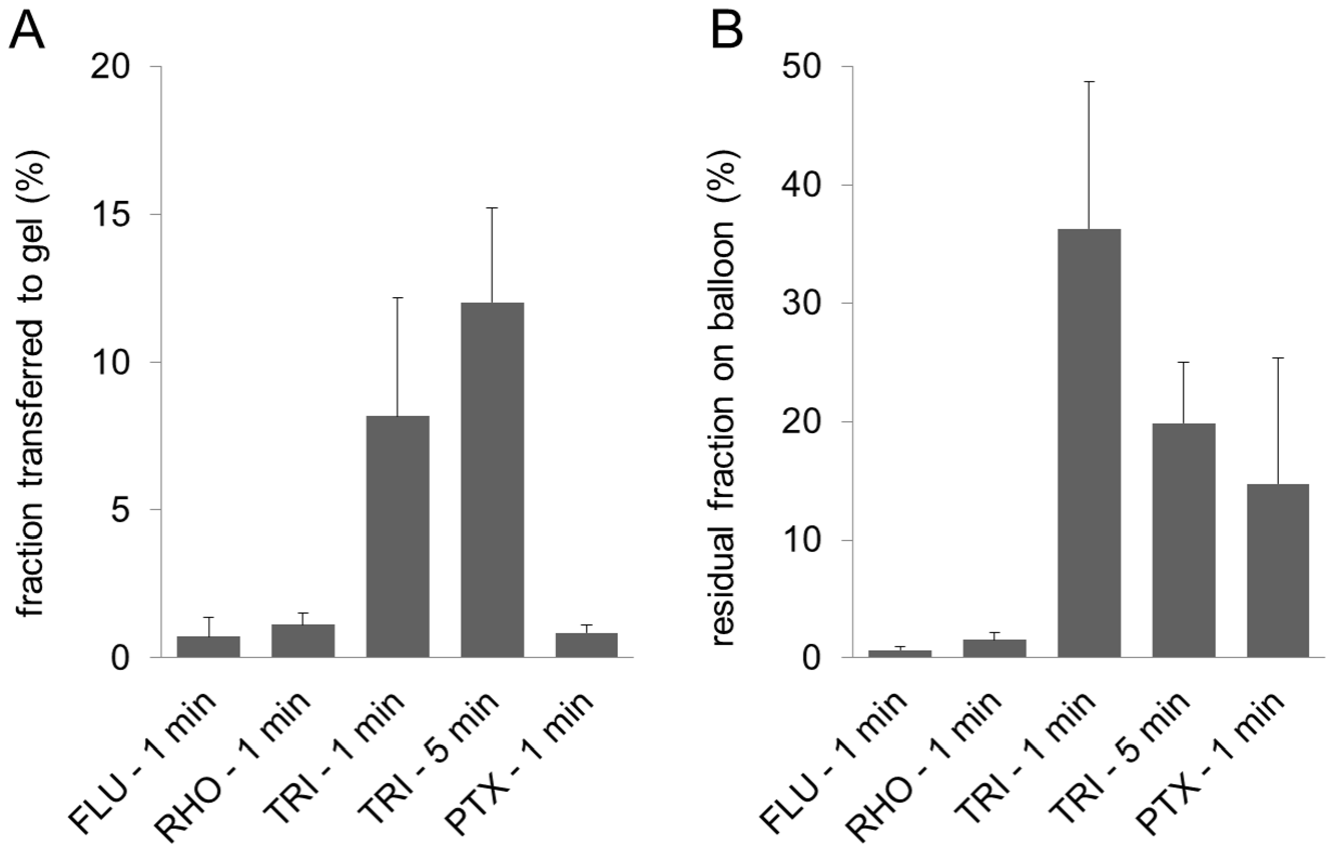
Drug transfer rate after simulated passage. Substance fraction transferred to the gel (A) and residual substance fraction on balloon (B) after advancement of the coated balloon through the model coronary pathway, FLU  =  fluorescein sodium, RHO  =  rhodamine B, TRI  =  triamterene, PTX  =  paclitaxel, time in min indicating the time span the balloon was in contact with the perfusion liquid phosphate buffered saline pH 7.4 prior to expansion into the gel for 1 min, means of n = 3±SD.

The results of drug transfer testing and the residual fractions remaining on the balloon after advancement through the perfused coronary artery model and expansion into the gel are shown in figure 5. In this case, the smallest transferred fractions were observed for FLU and PTX with transfer rates below 1% (0.7%±0.6% and 0.9%±0.3%, respectively) and RHO with 1.1%±0.4%. TRI-coated balloons achieved higher transfer rates with an average of 8%±4% after 1 min perfusion and 12%±3% after 5 min of perfusion. FLU- and RHO-coated balloons also possessed very small residual drug loadings after the experiments with 0.7%±0.6% and 1.5%±0.7%. Thus, more than 97% of the drug was lost during the simulated advancement procedure. Higher residual drug fractions on the balloon were detected for PTX (15%±11%) and TRI (36%±12% after 1 min perfusion and 20%±5% after 5 min perfusion). During the 5 min perfusion experiments with TRI-coated balloons, different fractions of the model substance were detected in the 5 discrete media samples. In detail, 37%±4% of the overall detected drug were washed away during the first min of perfusion, 2.2%±0.6% during the second min, 2.5%±2.2% in min 3, 0.9%±0.1% in minute 4 and 9%±2% in the last min of perfusion.

**Figure 6:**
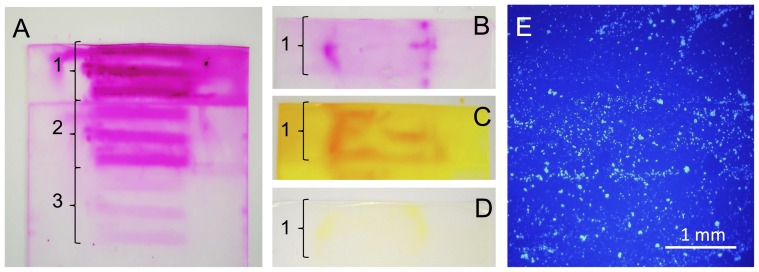
Visualization of drug distribution after transfer. Representative photographic (A–D) or fluorescence microscopic (E) images of gel films after 1 min expansion of rhodamine B-coated (A, B), fluorescein sodium-coated (C, D) or triamterene-coated (E) balloons into the film without (A, C) prior incubation or after advancement through the model coronary artery pathway including perfusion with phosphate buffered saline pH 7.4 for 1 min (B, D, E), braces and respective numbers indicating the number of coils of the gel film (first coil in direct contact with balloon).

Figure 6 exemplarily shows unwound gel films after expansion of model substance-coated balloons with or without prior advancement through the perfused model coronary artery pathway. In figure 6 A the first three layers of the gel film (layer 1 in direct contact with balloon, layers as indicated by numbered braces) are depicted, whereas in figure 6 B – D only the first layer is shown. In the samples not previously incubated, patterns can be observed in the gel film, indicating regions with higher model substance load. In the case of RHO (figure 6 A) this pattern in the form of 3 parallel bars can be observed through all of the 3 depicted layers of the gel film. The gel films obtained by expansion of FLU- and RHO-coated balloons that were previously incubated show markedly less coloration, distinct patterns were not observed. Coloration was, however, more intense at the parts of the films which were in contact with the proximal and distal end of the balloon. In the case of the TRI coatings, fluorescence microscopy only allowed for the imaging of much smaller portions of the gels (figure 6 E). Diffusely located particles can be observed along the film surfaces. Distinct distribution patterns were not observed.

## Discussion

Very little information on in vitro drug release and transfer from DCB has been published until to-date. However, with regard to the large variability which is to be expected in vivo due to different patient and lesion characteristics, standardized and reproducible in vitro test systems to characterize device performance under biorelevant conditions are highly desirable. Kelsch et al. [19] have published results regarding drug loss upon passage of a blood filled hemostatic valve and guiding catheter and subsequent immersion in blood for 1 min. Drug losses from DCB coated with PTX in combination with urea or iopromide amounted from 26%±3% to 36%±11%. Recently, Petersen et al. [20] described the use of an anatomic model according to ASTM F2934-07 to simulate the implantation procedure for DCB testing, similar to the model coronary artery pathway used for this study. Petersen et al., however, used a silicone tube as a model stenosed vessel opposed to the diffusible hydrogel used in this study. In their study using 50% cetylpyridinium salicylate in the coatings, PTX loss amounted to approximately 28%±12%.

In the study presented here, drug release and transfer from DCB were determined using a perfused model coronary artery pathway in combination with a gel film simulating the vessel wall as the target organ in this setup. A rolled up gel film has been used by our group before to examine model substance release from DES [18]. The main advantage of this setup is that it allows for the examination of spatial distributions in the unwound samples. Calcium alginate films are diffusible hydrogels and their size and shape can be assimilated to the situation in vivo. However, they lack many features of the vessel wall tissue including specific binding sites for certain molecules, such as PTX, which influence in vivo distribution [21]. Also, they are not yet optimized with regard to certain physicochemical parameters, such as diffusion and partition coefficients. Therefore, the used hydrogel films are only a very simplified simulation of the vessel wall and accurate prediction of the in vivo behavior of DCB cannot be expected from studies using this setup. The analysis of hydrogel films after DCB expansion may, however, be suitable to provide an idea of initial distributions of the transferred substances which are expected to be most relevant with respect to DCB. Efforts have been made to include lipophilic domains in calcium alginate hydrogels used for DES testing to further adapt the test setup to the situation in vivo, but this did not markedly affect release and distribution from DES [22].

Drug transfer from DCB to the simulated vessel wall may occur via two different ways. On the one hand, drug may dissolve upon contact with the hydrogel film and diffuse into the film. This was most likely the major way of transfer from the FLU and RHO coatings, since these drugs are well soluble in water. In this case, the extent of drug transfer will be determined by the rate of dissolution into a liquid film providing only a small volume. The resulting concentration gradient at the interface forces the transport by diffusion. Since both substances possess good equilibrium solubility, it may be speculated that the higher transfer rate of FLU (figure 4) is caused by a faster dissolution of the substance. Alternatively to drug transfer to the simulated vessel wall via dissolution and diffusion, particulate drug may be transferred due to mechanical forces acting on the coating during balloon expansion against the gel film. This was most likely the case with the TRI and PTX coatings. Both substances are characterized by very low saturation solubility and low dissolution rate [23], [24]. Also, the coatings contained particles (TRI, coating from suspension) and crystals (PTX, see figure 2) which may possibly split off during balloon expansion. Crystallization is a phenomenon often observed with PTX-containing balloon coatings, and the degree of crystallization has been proposed to impact on the clinical outcome of the treatment [25].

The extent of drug transfer in the case of nearly insoluble coatings must be expected to be mainly determined by the degree of adherence of the coating to the balloon surface and the adherence to the acceptor compartment (in this case the hydrogel). When comparing the drug transfer of the different model substances and PTX in the non-perfused setup, it becomes clear, that no assumptions regarding the transferred fraction can be drawn from the preferred transfer mechanism (dissolution and diffusion vs. particle transfer). Both methods may lead to the transfer of large fractions (FLU 78%±4%, PTX 45%±8%) or smaller fractions (RHO 24%±8%, TRI 14%±2%) depending on the physicochemical properties of the coatings and the incorporated substances. The images of the films after FLU- and RHO-coated balloon expansion support the assumption that these substances were predominantly transferred via dissolution and subsequent diffusion, since particles were not visible on the films. Contradictory, images of films after TRI-coated balloon transfer testing showed particulate substance. However, the model substances FLU and RHO are highly water soluble. Potential drugs are expected to possess much lower solubilities. Furthermore, the volume available for dissolution and the time for transfer are very short which potentially limits drug transfer via dissolution. Accordingly, particle transfer is much more likely to be the relevant route of drug transfer in vivo.

Especially for the coatings with high transfer rates in the simulated vessel wall in the non-perfused setup, high substance losses during the implantation procedure due to the onset of dissolution in the fluid filled guiding catheter and blood vessels and mechanical stress during the passage must be expected. The model coronary pathway and the related implantation procedure were established to simulate DCB advancement to the site of delivery in an in vitro setup. A perfusion time of 1 min in the model coronary artery pathway was chosen to simulate a fairly quick and uncomplicated implantation procedure. In our experiments approximately 20 s were necessary to advance the balloon along the guidewire towards the resting position. In clinical practice, an additional time span will be required in order to verify the correct positioning of the balloon via fluoroscopy prior to expansion. Since TRI-coated balloons possessed the highest residual loading after the advancement and subsequent expansion, an alternative perfusion time of 5 min simulating a more complex advancement procedure was also tested. Comparing the results with and without advancement through the model coronary artery pathway (figure 4 and 5 A) it is evident, that the simulated implantation procedure had a great impact on the transferred fraction from FLU-, RHO- and PTX-coated balloons with a multiple reduction in the transfer rate down to approximately 1%. For TRI coatings showing the lowest transfer rate in the non-perfused setup with 14%±2%, only a much smaller reduction in the transferred fraction was observed (8%±4% after 1 min, 12%±3% after 5 min of perfusion). In combination with the data for residual drug fraction on the balloon (figure 5 B), it must be assumed, that 1 min of perfusion in combination with possible abrasion during the model passage was sufficient to dissolve most of the FLU and RHO from the coatings, so that only a small fraction of the initial load remained for transfer. For the PTX coatings, a fraction of 84%±10% was lost during the simulated passage, but of the remaining PTX on the balloon only a small fraction (approximately 5% corresponding to 0.9% of initial load) was transferred upon expansion into the gel film compared to a fraction of 45% when transferred without prior advancement through the model. The reason for this is not entirely clear. Part of the non-transferred fraction may be drug located at the conical balloon shoulders which do not get in contact with the gel. Drug located here is also likely to remain for the largest part undissolved in the case of PTX and TRI during the short contact times with the perfusion media. Additionally, preceding wetting of the coating during the passage of the model coronary artery pathway might influence the transfer rate.

Compared to results reported by Kelsch et al. [19] the drug losses observed in this study were much higher. This may on the one hand be due to different coating morphologies. Different clinical studies have shown the superiority of PTX-coated balloons over uncoated balloons or PTX co-administration with a contrast agent [26]–[28]. However, it has also been shown that excipients may play a major role regarding the prevention of drug loss and the optimization of drug transfer. In further in vitro studies using the developed drug transfer model, additives should be included in the coatings to improve the PTX deliverability [29]. The used solvent has also been reported to impact on the clinical outcome [30]. This might also be associated with the crystallization behavior of PTX which may be different with solvents of different vapor pressure. On the other hand, the mechanical stress and onset of dissolution may be stronger in the perfused model coronary artery pathway simulating the in vivo DCB advancement compared to a blood filled hemostatic valve and guiding catheter and subsequent in vitro immersion in blood. In the animal study performed by Kelsch et al., 10 – 13% of the initial PTX load remained on the balloon after application in a porcine implantation model [19] which is comparable to the residual PTX fraction of 15% observed here. Scheller et al. [31] estimated a loss of up to 90% of the dose in vivo. In this context the results obtained regarding the wash-off of TRI from the coated balloon during 5 min perfusion are also very interesting. The largest fraction of the drug was detected in the perfusion media in the first minute of perfusion during which the balloon was advanced to the resting position. During the following minutes the balloon was retained in this position and fairly small losses occurred. At the very end of the experiment during the last seconds of perfusion the balloon was further advanced out of the model passing the last curve of the simulated artery pathway. The drug fraction contained in the respective media samples was higher compared to the previous media samples. This data suggests, that not the time of perfusion but the mechanical stress acting on the balloon during advancement may be the main force leading to drug loss in case of drug formulations with a low saturation solubility and low dissolution rate.

One of the often stated advantages of DCB over DES is the potentially homogenous drug delivery to the vessel wall [1], [4], as opposed to a very spatially defined delivery due to release from the comparably narrow stent struts (surface coverage of less than 20% [4]), as reported by Hwang et al. [32]. Nevertheless, the images of the films obtained after balloon expansion suggest that inhomogeneous distributions may also result from DCB delivery. Irrespective of differences in the distribution caused by irregularities of the treated vessel segments, homogenous distributions in the simulated vessel wall can only result, if the coating is homogenously distributed along the balloon surface at the moment of balloon expansion. This seems rather unlikely in the case of the DCB prepared for this study when taking into account the image of the separated coated balloon foil (figure 3 A). In this case, the coating was inhomogeneously distributed over the surface with the highest concentrations being located near the crest of the folds. This finding is in contrast to the common assumption that high concentrations are located within the folds and lower concentrations being located at the outer balloon [5], thus preventing washout during the advancement to the site of application. Whether the coating liquid can access the folds will, among other factors, be dependent on the folding technique and the resulting tightness of the folding. It can be assumed that other balloon foldings may lead to different coating distributions. Ruebben et al. [33] also reported on the primary location of PTX on the outer edges of the folds of CE-marked DCB including the clinically well-established SeQuent® Please DCB (B. Braun Melsungen AG, Germany). The observed location of the main part of the coating at the crests of the folds and on the outer surfaces in the folded state in the experiments presented here is in accordance with the observed distribution of the transferred drug. The three distinct intensively colored bars in figure 3 B are obviously caused by the coating located on the outside and near the crests of the folds. After advancement through the modeled coronary artery pathway the model substance located on the outside was washed away and a very low fraction is transferred, of which a greater portion was located at the distal and proximal shoulder of the balloon.

## Conclusion

Drug-coated balloons are a promising alternative to drug-eluting stents for some indications. Until to-date, the performance of these products is however not fully understood. The developed in vitro test setup employing a hydrogel film can be used to examine spatial distributions of transferred colored or fluorescent substances in the gel films and thus help to elucidate dosage form behavior under conditions adapted to the in vivo procedure. Besides the solubility of the substance which is to be administered via DCB, the coating morphology seems to be of greatest importance. It seems crucial to carefully design coatings to avoid vast drug losses during the advancement to the site of expansion while at the same time allowing for sufficient transfer upon expansion against the vessel wall. The results of the study presented here further indicate that using a micro-pipetting technique with tightly folded balloons may lead to inhomogeneously distributed coatings with little coating located deep within the folds. This finding, which is contradictory to common assumptions about this dosage form, emphasizes the necessity to further characterize device performance in vitro.
